# High-Performance and Water Resistant PVA-Based Films Modified by Air Plasma Treatment

**DOI:** 10.3390/membranes12030249

**Published:** 2022-02-22

**Authors:** Xin Rao, Qi Zhou, Qin Wen, Zhiqiang Ou, Lingying Fu, Yue Gong, Xueyu Du, Chunqing Huo

**Affiliations:** 1 Hainan Provincial Fine Chemical Engineering Research Center, Hainan University, Haikou 570228, China; 19085216210034@hainanu.edu.cn (X.R.); qduzhouqi@163.com (Q.Z.); wenqin_hg@hainanu.edu.cn (Q.W.); ouzhiqianghu@163.com (Z.O.); fulingyinghainan@163.com (L.F.); 13503529196@163.com (Y.G.); 2 Hainan Provincial Key Lab of Fine Chemistry, Hainan University, Haikou 570228, China; 3 School of Materials Science and Engineering, Hainan University, Haikou 570228, China

**Keywords:** air plasma, PVA film, surface modification, water resistance, crosslinking

## Abstract

Plasma treatment is considered a straightforward, cost-effective, and environmental-friendly technique for surface modification of film materials. In this study, air plasma treatment was applied for performance improvement of pure PVA, cellulose nanocrystal (CNC)/PVA, and CNC/oxalic acid (OA)/PVA films. Compared with the original performance of pure PVA, the mechanical properties and water resistance of air plasma treated films were greatly improved. Among them, the CNC/OA/PVA film treated by three minutes of air plasma irradiation exhibits the most remarkable performance in mechanical properties (tensile strength: 132.7 MPa; Young’s modulus: 5379.9 MPa) and water resistance (degree of swelling: 47.5%; solubility: 6.0%). By means of various modern characterization methods, the wettability, surface chemical structure, surface roughness, and thermal stability of different films before and after air plasma treatment were further revealed. Based on the results obtained, the air plasma treatment only changed the surface chemical structure, surface roughness, and hydrophobicity, while keeping the inner structure of films intact.

## 1. Introduction

To effectively alleviate the global environmental issue of plastic pollution, the development of biodegradable polymers has received tremendous attention in recent years. As a typical representative of semi-crystalline polymers, polyvinyl alcohol (PVA) is derived from the alcoholysis of polyvinyl acetate and its prevailing industrial applications are primarily ascribed to its remarkable properties in terms of biodegradability, biocompatibility, film-forming, solvent resistance, etc. [1]. Although PVA serves as a promising alternative to petroleum-based plastics, its application fields are still restricted by its poor water tolerance. The swelling phenomenon of PVA films inevitably occurs owing to the incorporation of water molecules and hydroxyl groups on the PVA molecular chains via strong hydrogen bonds, which consequently impairs the mechanical properties of the film products [2]. 

Therefore, proper structural modification is highly desired to improve the water resistance of PVA-based films. Traditionally, the modification of PVA films could be carried out by physical or chemical approaches [3]. Physical modification is featured as its relatively simplified preparation process and the establishment of hydrogen bonds between the reinforcing filler and PVA matrix normally accounts for the reason of its improved properties. To date, a large number of reinforcing fillers have been attempted to further improve the performance of PVA films, such as cellulose derivatives [4], modified starch [5], graphene [6], chitosan [7], etc. It is necessary to mention that although the reinforced network of hydrogen bonds does boost the mechanical properties to a large extent via effective stress transfer, it is still vulnerable if exposed to an aqueous or a high-humidity environment. 

Chemical modification, on the other hand, could essentially resolve the poor water resistance by building up stable covalent bonds between fillers and the PVA matrix with the participation of a cross-linker. As a result, the major performance of PVA-based films regarding mechanical properties, water resistance, and thermal stability could be promoted. Traditional chemical cross-linkers had been reported as glyoxal [8], glutaraldehyde [9], diisocyanate [10], etc. However, these aldehydes are deemed as potential toxic additives existing in the ultimate film products if they could not be completely reacted. In the context of green and sustainable chemistry, green cross-linkers are emerging as competitive alternatives known as succinic acid [11], malic acid [12], tartaric acid [13] and other types of polyacids. Although polyacids themselves are regarded as environment-friendly reagents, their crosslinking processes commonly should be assisted by relatively harsh conditions (e.g., strong acids as catalysts, high crosslinking temperature, etc.) 

Hence, it is still necessary to find a more environment-friendly modification technique for the preparation of water-tolerant PVA-based films. Recently, plasma treatment has received growing attention for its remarkable capacity in film surface modification. Compared with other film modification methods, plasma treatment has been highlighted by its low energy consumption, cost-effectiveness, and especially the fairly low output of byproducts or wastes [14]. As the fourth state of matter, plasma is generated from the ionization of specific gaseous molecules via dissociation of their intrinsic chemical bonds to form a collection of ions, electrons, free radicals, etc. [15,16]. Later, these highly reactive charged or neutral species would target on the surface of treated material during plasma irradiation and trigger scission and crosslinking of surface atoms and functional groups. Briefly, plasma treatment only functions on the surface of the material to cause physical etching and chemical reactions (e.g., crosslinking, grafting, etc.) without the assistance of additional initiators or reagents, while the inner characteristics of films still remains intact [17,18].

Relevant research has therefore been carried out to promote the utilization of plasma treatment in the field of film modification. For instance, Dalei et al. applied low temperature argon plasma to modify the surface of cellulose/PVA biocomposites, which ultimately resulted in increased hydrophilicity and antimicrobial activity [19]. Yang et al. reported to prepare argon plasma treated PVA/starch blends with improved mechanical and thermal properties [20]. However, detailed changes in the surface structure and surface chemical-bonding state of PVA-based films after plasma treatment still need to be deepened and further elucidated.

In this study, a more cost-effective air plasma treatment was applied to disclose its potential benefits on integral performance enhancement of three different types of PVA-based films, namely pure PVA film, cellulose nanocrystal (CNC)/PVA blend film, and CNC/oxalic acid (OA)/PVA crosslinked film. After regulation of different plasma treatment durations, all test specimens had been analyzed in terms of mechanical properties, water resistance, and surface wettability. The variation of chemical bonding state and functional groups on the surface of the films before and after air plasma treatment had been emphatically revealed by XPS and FTIR. In addition, the surface morphology, characteristic X-ray patterns, and thermal stability of different PVA-based films had also been characterized by modern analytical techniques including AFM, XRD, and TGA. 

## 2. Materials and Methods

### 2.1. Materials

Highly purified cellulose powder (99.5%) was purchased from North Century Cellulose Material Co., Ltd. (Xuzhou, China). PVA (degree of polymerization: 1700; degree of alcoholysis: 98–99% (mol/mol)) was purchased from Aladdin Co., Ltd. (Shanghai, China). Oxalic acid (99.5 wt%) and ethanol were supplied by Macklin Co., Ltd. (Shanghai, China). Besides, sulfuric acid (95.0–98.0 wt%) was provided by Xilong Science Co., Ltd. (Guangzhou, China). All chemicals used were of analytical pure grade and deionized water was used for all experiments.

### 2.2. Preparation of Cellulose Nanocrystals (CNCs)

10 mL of 64 wt% aqueous sulfuric acid solution and 1 g of cellulose fine powder were added into a 50 mL conical beaker. The acid hydrolysis reaction was performed under 50 °C for 1 h with magnetic stirring. Subsequently, the reaction was quenched by the addition of excess deionized water. The resultant residue obtained after centrifugation was further dialyzed for three days. Later, the purified suspension was transferred to a double-layer glass beaker and sonicated under 1200 W for 20 min by a JY98-IIIL ultrasonic cell crusher (Zhejiang Declair Instruments Co., Ltd., Hangzhou, China) at 4 °C. The CNC suspension was then acquired and stored in a refrigerator at 4 °C for future use.

### 2.3. Preparation of Pure PVA Film, CNC/PVA Blend Films, and CNC/OA/PVA Cross-Linked Films 

Pure PVA film was prepared by dissolving 23.0 g of PVA into 100 mL deionized water at 100 °C. After that, the solution was cooled at room temperature and set still for 3 h to expel air bubbles. The film was then fabricated by using a KW-4A benchtop spin coater (Institute of Microelectronics, Chinese Academy of Sciences, Beijing, China). In short, the PVA solution was gently added onto a clean square polyethylene substrate (15 cm × 15 cm) with a rotating speed at 400 rpm for 36 s. The spin-coated substrate was then dried in an oven at 50 °C for 24 h. Subsequently, the pure PVA film was peeled off from the substrate and kept in a sealed plastic bag for future use. 

CNC was served as a reinforcing filler in preparation of CNC/PVA blend film and its dry weight was formulated as 3% with respect to the dry weight of PVA. Briefly, a prescribed amount of CNC suspension was pre-dispersed in 100 mL deionized water under sonication. Later, 23.0 g of PVA was charged into the suspension and the whole system was treated at 100 °C with magnetic stirring until PVA particles were completely dissolved. The suspension was cooled at room temperature and set still for 3 h to remove air bubbles. The subsequent steps were the same as for the preparation of pure PVA film.

The preparation process of CNC/OA/PVA cross-linked film was briefly described as follows. First, a certain amount of CNC suspension was pre-dispersed into 100 mL deionized water with the assistance of sonication. The dry weight of CNC was controlled as 3% based on the dry weight of PVA. Later, 6.9 g of OA and 23.0 g of PVA were supplemented into the above suspension and the entire system was heated up to 100 °C with magnetic stirring until the PVA was completely dissolved. The resultant mixture was cooled at room temperature and set still for 3 h to remove air bubbles. The subsequent spin coating procedure was the same as described for the preparation of pure PVA film. Finally, the resultant product was further treated at 120 °C for 0.5 h to obtain CNC/OA/PVA cross-linked films. The thickness of all film specimens were controlled at 0.1 mm. 

### 2.4. Air Plasma Treatment 

The air plasma treatment of PVA-based films were performed by using a SPA-2800 air plasma cleaner (Guangdong Dainty Intelligent Technology Co., Ltd., Dongguan, China). The main operation parameters are described as follows: voltage (220 V), power (580–620 W), air pressure (0.1–0.3 MPa). Each type of PVA-based films was treated by three different time intervals (i.e., 1 min, 3 min, and 5 min). The pure PVA films exposed to 1 min, 3 min, and 5 min of air plasma were labeled as PPVA-1, PPVA-3, and PPVA-5, respectively. Likewise, the CNC/PVA blend films treated by 1 min, 3 min, and 5 min of air plasma were labeled as CNC/PPVA-1, CNC/PPVA-3, and CNC/PPVA-5, respectively. A similar labeling rule was also applied to air plasma-treated CNC/OA/PVA films.

### 2.5. Characterization

#### 2.5.1. X-ray Photoelectron Spectroscopy

The surface chemistry and elemental composition of different films were analyzed by a 250Xi X-ray photoelectron spectrometer (Thermo Fisher Scientific, Waltham, MA, USA), equipped with a monochromated Al-Kα source (1486.6 eV). The spectra were recorded in a background pressure of 1 × 10^−10^ mbar. Besides, the pass energy was set as 100 eV for survey spectra (a step of 1 eV) and 20 eV for high resolution spectra (a step of 0.1 eV). All XPS spectra acquired were further processed by Avantage software. 

#### 2.5.2. Fourier Transform Infrared Spectroscopy (FTIR)

FTIR was carried out by a Bruker Nicolet iS50 spectrophotometer (Karlsruhe, Germany) with an attenuated total reflectance (ATR) attachment. The spectra were recorded in the range from 4000 cm^−1^ to 500 cm^−1^ at a resolution of 4 cm^−1^.

#### 2.5.3. Atomic Force Microscopy (AFM) 

The surface morphology of films was investigated by using a Dimension Icon atomic force microscope (Bruker, Karlsruhe, Germany). The root mean square roughness (Rq) was measured by using NanoScope Analysis software. 

#### 2.5.4. Water Resistance

The water resistance of different films was evaluated including their individual degree of swelling and solubility. All samples were pre-cut into the squares of 2.0 cm × 2.0 cm and immersed in deionized water at room temperature for 24 h. The samples were then removed and immediately dried with dust-free tissues to remove surface moisture. Later, the swelled samples were dried in an oven at 50 °C until the weight was constant. The original dry weights of films and the weights before and after oven drying for calculating the degree of swelling and solubility were recorded according to Equations (1) and (2) as follows:

(1) $$\mathrm{Degree}\;\mathrm{of}\;\mathrm{swelling}=\frac{\;m_{b}-m_{a}}{m_{a}}\times 100\%$$

(2) $$\mathrm{Solubility}=\frac{\;m_{a}-m_{c}}{\;m_{a}}\times 100\%$$

where *m_a_* is the original dry weight of test film, g; *m_b_* is the weight of the swelled film after removing its surface moisture, g; and *m_c_* is the dry weight of the film re-dried after soaking in deionized water for 24 h.

#### 2.5.5. Mechanical Properties

The tensile strength and Young’s modulus of different films were determined by an Instron 3343 long travel extensometer, using a load of 1 KN at a strain rate of 10 mm/min. The sample dimension was sized by 1 cm (width) × 10 cm (length) and a minimum of five test runs were carried out for each specimen prior to the calculation of average value and deviation. All specimens were tested at an ambient temperature (25 °C) and 80% relative humidity. 

#### 2.5.6. Water Contact Angle 

The water contact angles of different films were measured by an SL200KB optical contact angle/interface tensiometer (Kino Industry Co. Ltd., Boston, MA, USA). The images of water droplets were taken 3 s after placing the droplets, and all the measurements were performed at 25 °C.

#### 2.5.7. Thermogravimetric Analysis

The thermal stability of different films were determined by a NETZSCH STA 449 F5 thermogravimetric analyzer (NETZSCH, Selb, Germany). Each sample was heated from 30 °C to 600 °C at a rate of 10 °C/min and the flow rate of carrier gas (N_2_) was set as 20 mL/min.

#### 2.5.8. X-ray Diffraction Analysis

The crystal characteristics of different films was investigated by a Smart Lab X-ray diffractometer (Rigaku, Tokyo, Japan). Each measurement was scanned from 5° to 30° at a rate of 5°/min.

## 3. Results and Discussion

### 3.1. Mechanical Properties

**Figure 1:**
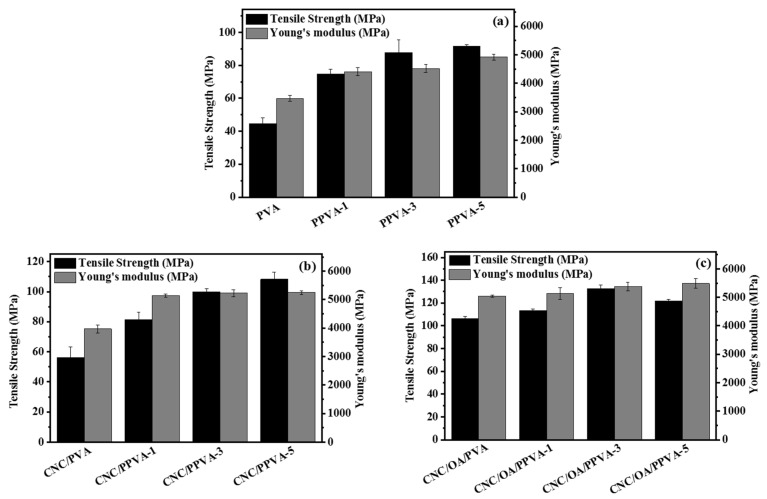
Tensile strength and Young’s modulus of different films without or with air plasma treatment. (**a**) PVA and its air plasma-treated films (PPVA-1, PPVA-3, and PPVA-5 are referring to the PVA films exposed to air plasma irradiation by 1 min, 3 min, and 5 min, respectively.); (**b**) CNC/PVA and its air plasma-treated films (CNC/PPVA-1, CNC/PPVA-3, and CNC/PPVA-5 are referring to the CNC/PVA films exposed to air plasma irradiation by 1 min, 3 min, and 5 min, respectively.); (**c**) CNC/OA/PVA and its air plasma-treated films (CNC/OA/PPVA-1, CNC/OA/PPVA-3, and CNC/OA/PPVA-5 are referring to the CNC/OA/PVA films exposed to air plasma irradiation by 1 min, 3 min, and 5 min, respectively).

Film materials are subject to various external forces during practical use. If the major mechanical properties fail to meet the basic requirements, its application fields will be largely restricted. Tensile strength and Young’s modulus are two key performance indexes for evaluating the mechanical properties of films [21], and their corresponding values of different PVA-based films are shown in Figure 1.

For PVA and CNC/PVA films, the plasma treatment favors the enhancement of both tensile strength and Young’s modulus. Their respective values continuously increase following the increment of plasma irradiation time. The maximum values were achieved for PPVA-5 (tensile strength: 91.6 MPa; Young’s modulus: 4921 MPa) and CNC/PPVA-5 (tensile strength: 108.3 MPa; Young’s modulus: 5243.8 MPa) when 5 min of plasma irradiation was applied (Figure 1a,b). During plasma irradiation, the C–C and C–H bonds present on film surface are disrupted by reactive ionic species. As a result, the generated radicals can react with surface radicals or participate in surface cross-linking reactions, resulting in a remarkable increase in mechanical properties [22]. In addition, the moisture content of the specimen was further eliminated during plasma irradiation, which rendered the PVA molecular chains more tightly bound with each other via inter and intramolecular hydrogen bonds [23]. More carbonyl groups could be formed during plasma irradiation, also contributing to a more advanced network of hydrogen bonds. This may greatly improve the structural integrity of the film, enhance interfacial interactions between/among film components, and favor the stress transfer [24].

For CNC/OA/PVA and its air plasma treated films, the variation trend of Young’s modulus still remains consistent, as with the cases mentioned above. However, after reaching the maximum value of tensile strength (132.7 MPa) as in the case of CNC/OA/PPVA-3, its value started to decline with further extension of plasma irradiation time up to 5 min (Figure 1c). This may be due to the severe physical etching on the film surface caused by long-term plasma bombardment, which destroyed the original densely cross-linked surface structure of the film [25].

### 3.2. Water Resistance

**Figure 2:**
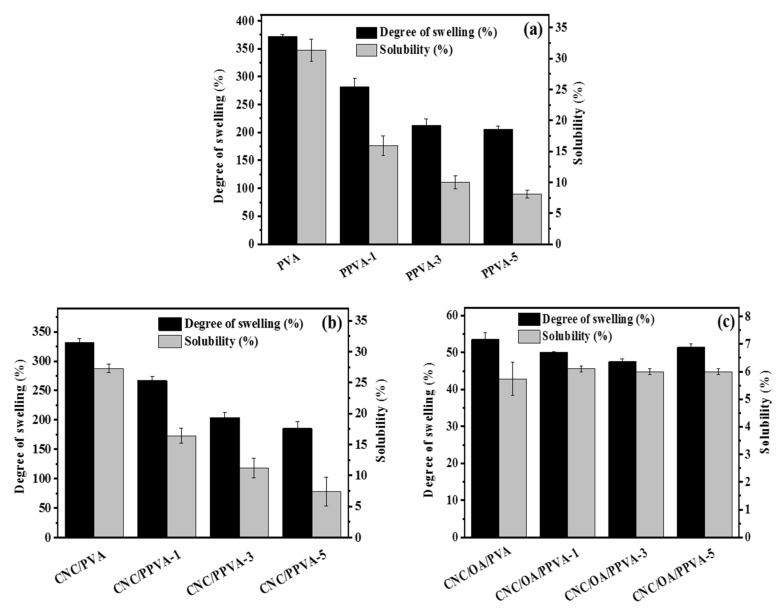
The degree of swelling and solubility of different films without or with air plasma treatment. (**a**) PVA and its air plasma-treated films; (**b**) CNC/PVA and its air plasma-treated films; (**c**) CNC/OA/PVA and its air plasma-treated films.

The improvement of water resistance for different films after air plasma treatment is demonstrated in Figure 2. It is noteworthy that both the degree of swelling and solubility of plasma-treated PVA and plasma-treated CNC/PVA films exhibit a continuous reduction trend when plasma irradiation time was extended from 1 min to 5 min. The minimum values of degree of swelling (185.6%) and solubility (7.4%) for CNC/PPVA-5 are even lower than those (degree of swelling: 205.8%; solubility: 8.1%) for PPVA-5 (Figure 2a,b). During the process of plasma treatment, the oxygen radicals take part in the formation of a crosslinking network on the film surface, which effectively prevents the invasion of water molecules [23]. Also, these reactive oxygen radicals favor the oxidation of hydroxyl groups on the film surface to carbonyl groups, which facilitates the formation of hydrogen bonds [26]. 

Ascribed to the inherent ester bond cross-linkages, the water resistance of CNC/OA/PVA film is more superior to that of CNC/PPVA-5 film. The degree of swelling and solubility of CNC/OA/PVA film are low up to 53.5% and 5.7%, respectively. It is noticeable that there is no evident change in solubility for CNC/OA/PVA films treated by different time intervals of air plasma. However, the degree of swelling rebounds gently to 51.4% (for CNC/OA/PPVA-5) after reaching its minimum value of 47.5% (for CNC/OA/PPVA-3) (Figure 2c). Generally, the plasma treatment did improve the water tolerance of CNC/OA/PVA film but to a minor extent. The highly reactive charged and neutral particles generated by plasma irradiation can readily oxidize hydroxyl groups and contribute to a more reinforced crosslinking network on the film surface, but the number of free hydroxyl groups in CNC/OA/PVA film is fairly limited, the majority of which had already been consumed for the establishment of ester bond cross-linkages. Moreover, those plasma particles mentioned above may still be inefficient to interrupt the inherent ester bond cross-linkages within a short time of plasma treatment. However, if the treatment time had lasted for 5 min (for CNC/OA/PPVA-5), the accumulated plasma etching could cleave the original ester bonds to some extent and eventually result in an increment of the degree of swelling. 

### 3.3. Surface Wettability

**Figure 3:**
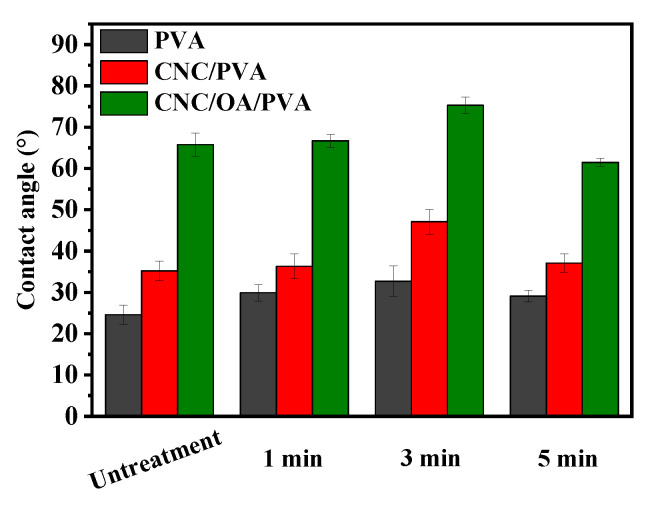
Contact angles of different films treated without or with air plasma.

The water contact angle is considered to be an important index to quickly assess the hydrophilicity or hydrophobicity of films. The higher the contact angle is, the more hydrophobic surface the film possesses. It is clear that the contact angle of the film with plasma treatment is higher than that of the untreated film (Figure 3). Furthermore, when the time of treatment is prolonged from 1 min to 3 min, the surface of test films turned out to be more hydrophobic, reflected by their increasing contact angles in different degrees. For each series of films, their corresponding highest contact angles could all have been acquired when 3 min of treatment time was applied. As mentioned before, the plasma treatment promotes the oxidation of hydroxyl groups into carbonyl groups that are more prone to form new hydrogen bonds. Therefore, the film surface turns out to be more hydrophobic, followed by a reduced amount of hydrophilic hydroxyl groups [26]. However, this increasing trend of surface hydrophobicity could not be well maintained if the time of plasma irradiation continues to proceed. When 5 min of irradiation was applied, the contact angles of all test films dropped to different extents. The contact angle of CNC/OA/PPVA-5 film (61.5°) is even lower than CNC/OA/PVA film (65.8°) (Figure 3). This result may be attributed to the excessive etching by air plasma, which destroys the hydrophobic structure by releasing more polar groups present on the film surface. 

### 3.4. Surface Chemical Study

**Figure 4:**
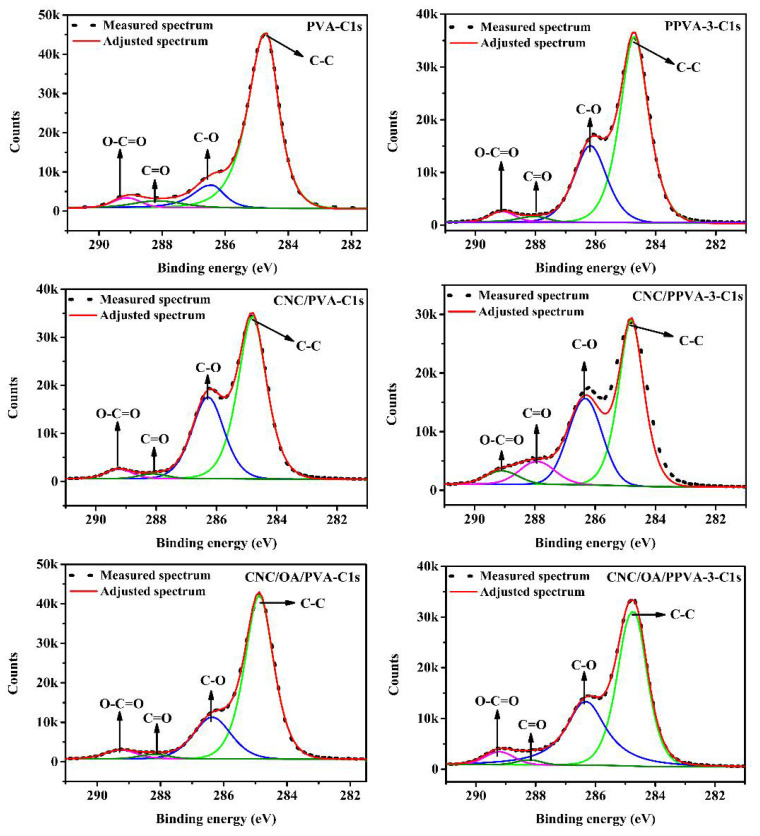
High resolution XPS spectra of C1s of different films without and with air plasma treatment.

To better elucidate the elemental composition and chemical changes of plasma modified film surfaces, XPS was applied and the relevant results are summarized in Table 1. According to the relative contents of C and O atoms from the film surface, the C/O ratios of plasma modified films are calculated and the results are conspicuously lower than those of untreated films. This implies the presence of more oxygen-containing groups existing on the film surface. Here, high resolution XPS spectra of C1s and O1s were recorded to release more information regarding chemical bonding relationships (Figure 4 and Figure S1). As shown in Figure 4, four deconvoluted peaks are observed for all three types of untreated films. Their respective binding energy was detected as 284.8 eV, 286.2 eV, 288.2 eV, and 289.1 eV, corresponding to C–C [27], C–O [28], C=O, and O–C=O [29], respectively. In addition, the O1s spectra of all six films were also resolved and fitted in the Supplementary Materials (Figure S1). It is interesting to note that there is only one peak referring to C–O (531.4 eV) [30], while two more deconvoluted peaks emerged at 530.2 eV (C=O) and 533.8 eV (O–C=O) in the spectrum of PPVA-3-O1s [31]. This directly verifies the presence of newly formed carbonyl and carboxylic groups after plasma treatment. It is necessary to point out that those groups (O–C=O and C=O) are inherent in untreated CNC/PVA and CNC/OA/PVA films, which explains the existence of their corresponding signals in the spectra of CNC/PVA-O1s and CNC/OA/PVA-O1s. After plasma treatment, however, the intensities of these peaks had been evidently enhanced. 

The relative contents of different chemical bonding states are listed in Table 2. According to the spectra of C1s, the relative contents of C–C bonds of plasma modified films are lower than those of their respective untreated films. This result is well supported by an earlier documented study that found that the original C–H and C–C bonds in PVA could be oxidized to C–O, C=O, and O–C=O groups after air plasma treatment [32]. In other words, the increasing relative contents of oxygen-containing groups results in the decrement of the relative content of C–C bonds. Besides, the effects of air plasma etching, caused by reactive oxygen radicals, may also destroy the surface chemical linkages e.g., C–C bonds and thus brings down its relative contents [18]. According to the spectra of O1s, the relative contents of O–C=O and C=O also increase as a consequence of air plasma treatment. A possible reaction mechanism for air plasma treatment of CNC/OA/PVA is proposed in Figure S2.

### 3.5. Surface Functional Groups

**Figure 5:**
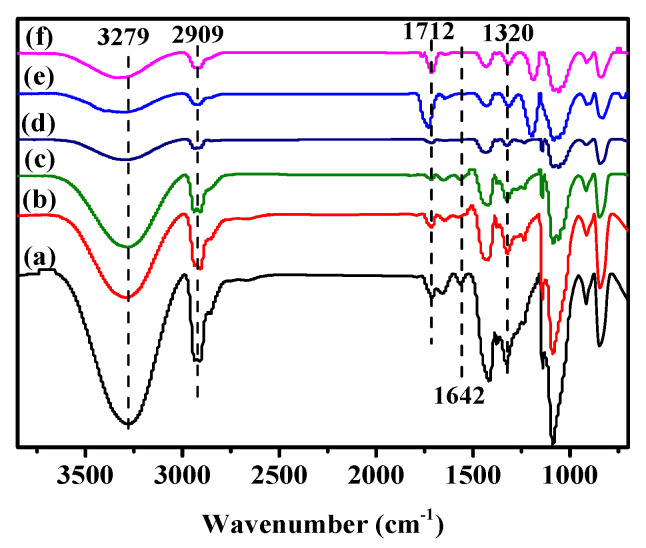
FTIR spectra of different films before and after air plasma treatment. (**a**) PVA film; (**b**) PPVA–3 film; (**c**) CNC/PVA film; (**d**) CNC/PPVA–3 film; (**e**) CNC/OA/PVA film; (**f**) CNC/OA/PPVA–3 film.

The FTIR spectra of different films before and after air plasma treatment are illustrated in Figure 5. In contrast to the spectra of untreated films, there are no new adsorption bands observed in the spectra of plasma treated films, indicating the air plasma treatment did not substantially change the bulk structure of the PVA-based films. Despite this, the plasma treatment did change the intensities of some major adsorption peaks. For instance, the intensities of adsorption bands around 3279 cm^−1^, referring to O–H stretching vibration, are significantly weakened for plasma treated films, which is due to the oxidation of hydroxyl groups to carbonyl and carboxylic groups during plasma treatment [24]. The reduction of hydroxyl groups is also evidenced by the decreased adsorption peak of O–H bending vibration at 1320 cm^−1^. Besides, the cleavage of C–H and C=C bonds during plasma treatment could also be reflected by the decreased intensities of C–H (2909 cm^−1^) and C=C (1642 cm^−1^) stretching vibration peaks. These types of cleavage also promote the formation of more reaction sites on the film surface [33]. 

### 3.6. Surface Morphology

**Figure 6:**
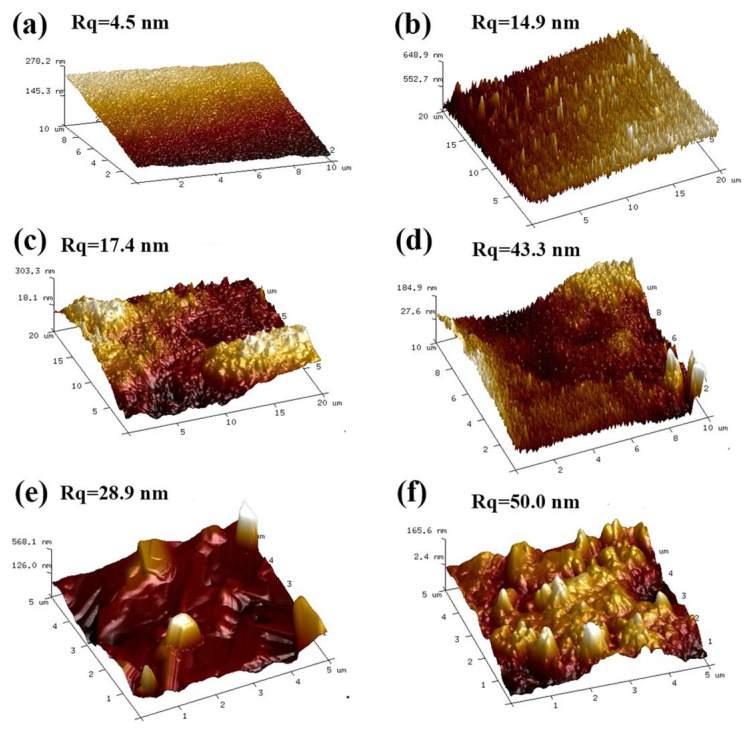
AFM images of different films before and after air plasma treatment. (**a**) PVA film; (**b**) PPVA-3 film; (**c**) CNC/PVA film; (**d**) CNC/PPVA-3 film; (**e**) CNC/OA/PVA film; (**f**) CNC/OA/PPVA-3 film.

The occurrence of surface etching is inevitable during plasma treatment due to the impact of high-energy and highly reactive particles targeted onto the film surface. Detailed structural information regarding surface morphology and roughness of different films before and after plasma treatment were characterized by atomic force microscopy (AFM) (Figure 6). As demonstrated in Figure 6, the surface of PVA, CNC/PVA, and CNC/OA/PVA films is relatively smooth and their corresponding surface roughness was analyzed to be 4.5 nm, 17.4 nm, and 28.9 nm, respectively. After 3 min of plasma treatment, the surface of modified films including PPVA-3, CNC/PPVA-3, and CNC/OA/PPVA-3 turned out to be more coarse and irregular, and more nano-structured columnar bumps could also be observed. Their corresponding surface roughness increased to 14.9 nm, 43.3 nm, and 50.0 nm, respectively. This significant increase in surface roughness for all treated films is attributed to the physical etching/ion bombardment by excited particles during plasma treatment [33,34]. According to related studies, increased surface roughness may also lead to high surface hydrophobicity [35], which is confirmed by the higher contact angles of three plasma treated films (Figure 3). And the surface roughness is greatly influenced by the power and time of plasma treatment [36]. 

### 3.7. Crystal Structure

**Figure 7:**
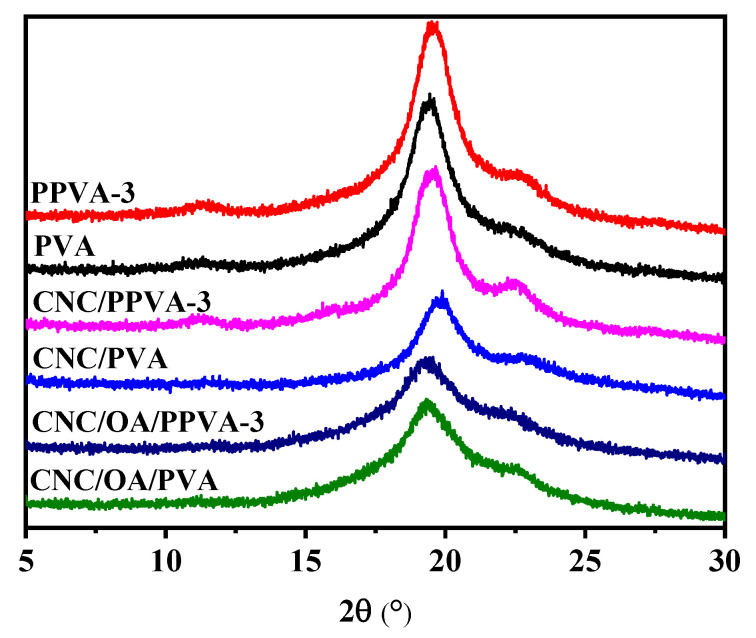
Effect of air plasma on X-ray diffraction patterns of different films before and after air plasma treatment.

The X-ray diffraction patterns of different films are shown in Figure 7. Characteristic diffraction signals of PVA are detected at 19.8° and 22.8° for all test films, which are originating to the 101 and 200 planes of semi-crystalline PVA, respectively [37,38]. Notably, there is no obvious difference in diffraction patterns of different films, indicating that air plasma treatment does not vary the bulk crystal structure of PVA, which is in line with the findings of a recent study [18]. Nevertheless, the air plasma irradiation did increase the signal intensities at 19.8°, which is probably attributed to the minor structural changes of PVA molecular chains by plasma irradiation [16].

### 3.8. Thermal Stability

**Figure 8:**
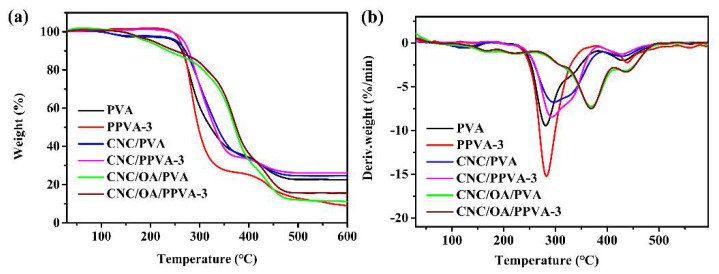
TGA (**a**) and DTG (**b**) thermographs of different films before and after air plasma trea–ment.

The effect of air plasma treatment on thermal stability of different films were evaluated by thermogravimetric analysis (Figure 8). As shown in Figure 8a, the weight loss occurred from 80 °C to 160 °C is due to the removal of free and bound moisture from samples. Later, two obvious weight-loss periods are observed for pure PVA film. The first period ranging from 190 °C to 380 °C is related to the dehydration of PVA sidechains and the second period ranging from 380 °C to 500 °C is ascribed to the degradation of PVA backbones [39]. For all test films, the temperature at 10% weight loss of each plasma-treated film is slightly higher than that of the corresponding untreated one (Table S1 in the Supplementary Materials). Furthermore, the onset temperature of decomposition (T_onset_) and the temperature of maximum weight-loss rate (T_DTGmax_) of major weight-loss stages of air plasma-treated films are fairly close to those of untreated films (Figure 8b and Table S2). Despite this, it is evident to note that the PPVA-3 film loses weight more readily than pure PVA film in the temperature range from 260 °C to 320 °C (Figure 8a,b). This is probably owing to the presence of partially cleaved C–C bonds and newly formed oxygen-containing groups on the film surface after air plasma treatment. Although CNC/PPVA-3 film is more thermally stable than PPVA-3 owing to the reinforcement of CNC, its weight-loss rate is still higher than that of CNC/PVA from 272 °C to 342 °C. It is interesting to note that the difference of thermal stability between CNC/OA/PVA and CNC/OA/PPVA-3 is negligible. In spite of the chemically modified surface by air plasma treatment, the inherent crosslinking network of ester bonds in CNC/OA/PVA film still takes a crucial role in keeping its relatively high thermal stability during thermogravimetric analysis. Besides, without the reinforcement of CNC (nano-filler) and/or crosslinking network, the residual mass (8.9%) of PPVA-3 film is far less than that (22.6%) of pure PVA film, while the residual weights of the other two plasma-treated films (namely, CNC/PPVA-3 and CNC/OA/PPVA-3) are in turn slightly higher than those of their respective untreated films (Table S1, Supplementary Materials). 

## 4. Conclusions

A green surface modification method by air plasma treatment was developed for preparation of a series of PVA-based films with remarkable water resistance and enhanced mechanical properties. After merely 3 min of air plasma irradiation, its modified product (CNC/OA/PPVA-3 film) possesses superior mechanical properties (tensile strength: 132.7 MPa; Young’s modulus: 5379.9 MPa) and water resistance (degree of swelling: 47.5%; solubility: 6.0%). The air plasma treatment only changes the chemical structure of the film surface by increasing surface roughness and the amounts of oxygen-containing groups while leaving the internal structure intact. The hydrophobicity of the film surface could also be effectively increased by selecting proper treatment time but lengthy times of plasma etching should be avoided. Therefore, these air plasma treated PVA-based films could be applied as green packaging materials with enhanced water resistance and mechanical properties.

## Figures and Tables

**Table 1:** Atomic composition and C/O ratios of different films without or with air plasma treatment.

Sample	C	O	C/O
PVA	79.5	20.5	3.9
PPVA-3	74.0	26.0	2.8
CNC/PVA	72.3	27.7	2.6
CNC/PPVA-3	67.5	32.5	2.1
CNC/OA/PVA	77.0	23.0	3.4
CNC/OA/PPVA-3	71.3	28.7	2.5

**Table 2:** Relative contents of different chemical bonding relationships in XPS-C1s and -O1s spectra.

Samples	Relative Contents (%)						
	C1s				O1s		
	C–C	C–O	O–C=O	C=O	C–O	O–C=O	C=O
PVA	81.4	10.6	3.6	4.4	100.0	–	–
PPVA-3	65.1	25.5	4.3	5.1	83.6	11.8	4.6
CNC/PVA	61.8	33.4	3.2	1.6	85.5	10.1	4.4
CNC/PPVA-3	51.9	33.7	9.4	5.1	64.5	19.6	15.9
CNC/OA/PVA	71.3	22.8	4.1	1.8	81.8	15.0	3.2
CNC/OA/PPVA-3	56.5	34.8	5.6	3.1	71.6	20.8	7.5

## Data Availability

Not applicable.

## Supplementary Materials

The following supporting information can be downloaded at: https://www.mdpi.com/article/10.3390/membranes12030249/s1, Figure S1: High resolution XPS spectra of O1s of different films without and with air plasma treatment; Table S1: The temperature at 10% weight loss and residual mass of different PVA-based films; Table S2: T_onset_ and T_DTGmax_ values of major weight-loss stages of different PVA-based films; Figure S2: A possible reaction mechanism for air plasma treatment of CNC/OA/PVA film. The groups/linkages before and after air plasma treatment are marked with red-dashed circles and in red font color, respectively.
