# Supplementary material for: High-Performance and Water Resistant PVA-Based Films Modified by Air Plasma Treatment
Source: Membranes (Basel). 2022 Feb 22;12(3):249. doi: 10.3390/membranes12030249 (PMC8951830; doi:10.3390/membranes12030249)
Supplement: Supplementary file 1 [file membranes-12-00249-s001.zip › membranes-1598774-supplementary.pdf]

Supplementary materials

High-performance and water resistant PVA-based films modified by air plasma treatment

Xin Rao [1,2], Qi Zhou [1,2], Qin Wen [1,2], Zhiqiang Ou [1,2], Lingying Fu [1,2], Yue Gong [1,2], Xueyu Du [1,2,*] and Chunqing Huo [3,*]

1 Hainan Provincial Fine Chemical Engineering Research Center, Hainan University, Haikou 570228, China; 1632110422@qq.com (X.R.); qduzhouqi@163.com (Q.Z.); wenqin_hg@hainanu.edu.cn (Q.W.); 895370225@qq.com (Z.O.); 821423846@qq.com (L.F.); 3466067419@qq.com (Y.G.)

2 Hainan Provincial Key Lab of Fine Chemistry, Hainan University, Haikou, Hainan 570228, China.

3 School of Materials Science and Engineering, Hainan University, Haikou, Hainan 570228, China.

* Correspondence: duxueyu@hainanu.edu.cn (X.D.); chunqinghuo@hainanu.edu.cn (C.H.)

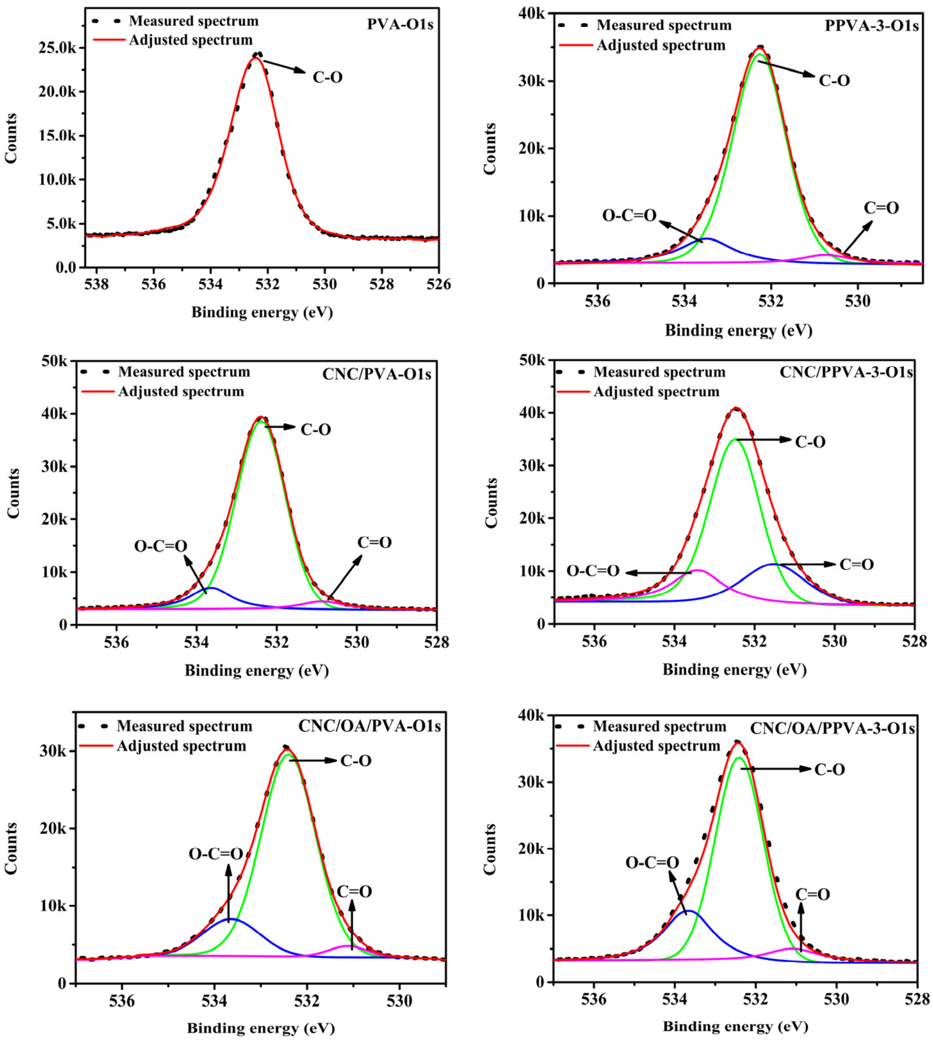

Figure S1. High resolution XPS spectra of O1s of different films without and with air plasma treatment.

Table S1. The temperature at 10% weight loss and residual mass of different PVA-based films.

Samples	Temperature at 10% weight loss (°C)	Residual mass (%)
PVA	264	22.6
PPVA-3	267	8.9
CNC/PVA	271	24.7
CNC/PPVA-3	278	26.1
CNC/OA/PVA	237	11.1
CNC/OA/PPVA-3	252	15.5

Table S2. T_{onset} and T_{DTGmax} values of major weight-loss stages of different PVA-based films.

Samples	Major weight-loss periods			
	The 1st period		The 2nd period	
	T_{onset} (°C)	T_{DTGmax} (°C)	T_{onset} (°C)	T_{DTGmax} (°C)
PVA	216	281	382	431
PPVA-3	216	282	382	440
CNC/PVA	207	297	389	429
CNC/PPVA-3	208	294	384	428
CNC/OA/PVA	259	369	414	436
CNC/OA/PPVA-3	259	369	413	437

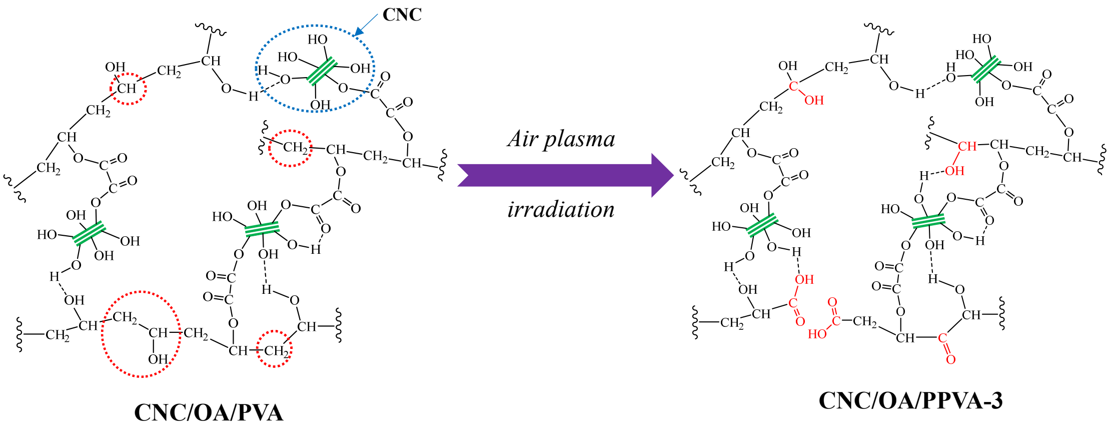

Figure S2. A possible reaction mechanism for air plasma treatment of CNC/OA/PVA film. The groups/linkages before and after air plasma treatment are marked with red-dashed circles and in red font color, respectively.
